# Application of a Reverse Genetic System for *Beet Necrotic Yellow Vein Virus* to Study *Rz1* Resistance Response in Sugar Beet

**DOI:** 10.3389/fpls.2019.01703

**Published:** 2020-01-17

**Authors:** Sebastian Liebe, Daniel Wibberg, Edgar Maiss, Mark Varrelmann

**Affiliations:** ^1^Department of Phytopathology, Institute of Sugar Beet Research, Göttingen, Germany; ^2^Genome Research of Industrial Microorganisms, CeBiTec, Bielefeld University, Bielefeld, Germany; ^3^Plant Virology, Department of Phytomedicine, Institute of Horticultural Production Systems, Leibniz University, Hannover, Germany

**Keywords:** beet necrotic yellow vein virus, mutation, resistance-breaking, reverse genetic system, *Rz1*, sugar beet, virus evolution

## Abstract

*Beet necrotic yellow vein virus* (BNYVV) is causal agent of rhizomania disease, which is the most devastating viral disease in sugar beet production leading to a dramatic reduction in beet yield and sugar content. The virus is transmitted by the ubiquitous distributed soil-borne plasmodiophoromycete *Polymyxa betae* that infects the root tissue of young sugar beet plants. *Rz1* is the major resistance gene widely used in most sugar beet varieties to control BNYVV. The strong selection pressure on the virus population promoted the development of strains that can overcome *Rz1* resistance. Resistance-breaking has been associated with mutations in the RNA3-encoded pathogenicity factor P25 at amino acid positions 67–70 (tetrad) as well as with the presence of an additional RNA component (RNA5). However, respective studies investigating the resistance-breaking mechanism by a reverse genetic system are rather scarce. Therefore, we studied *Rz1* resistance-breaking in sugar beet using a recently developed infectious clone of BNYVV A-type. A vector free infection system for the inoculation of young sugar beet seedlings was established. This assay allowed a clear separation between a susceptible and a *Rz1* resistant genotype by measuring the virus content in lateral roots at 52 dpi. However, mechanical inoculation of sugar beet leaves led to the occurrence of genotype independent local lesions, suggesting that *Rz1* mediates a root specific resistance toward BNYVV that is not active in leaves. Mutation analysis demonstrated that different motifs within the P25 tetrad enable increased virus replication in roots of the resistant genotype. The resistance-breaking ability was further confirmed by the visualization of BNYVV in lateral roots and leaves using a fluorescent-labeled complementary DNA clone of RNA2. Apart from that, reassortment experiments evidenced that RNA5 enables *Rz1* resistance-breaking independent of the P25 tetrad motif. Finally, we could identify a new resistance-breaking mutation, which was selected by high-throughput sequencing of a clonal virus population after one host passage in a resistant genotype. Our results demonstrate the feasibility of the reverse genetic system for resistance-breaking analysis and illustrates the genome plasticity of BNYVV allowing the virus to adapt rapidly to sugar beet resistance traits.

## Introduction

*Beet necrotic yellow vein virus* (BNYVV) is a member of the genus *Benyvirus* within the family *Benyviridae* (Gilmer et al., 2017). The virus is the causal agent of rhizomania disease in sugar beet, which is distributed worldwide (reviewed in McGrann et al., 2009) and causes a dramatic reduction in beet yield and sugar content. Severe disease symptoms including reduced size, wine-glass like shape, and necrosis of vascular tissue can be observed on infected taproots. Extensive proliferation of lateral roots is induced leading to a root beard, which is the characteristic symptom of the rhizomania disease. Systemic symptoms on leaves are characterized by vein yellowing and necrosis, but can be seldomly observed in the field. The virus is transmitted by the soil-borne plasmodiophoromycete *Polymyxa betae* that infects the root tissue of sugar beet plants (Keskin, 1964; Tamada and Kondo, 2013). Resting spores can survive in the soil for decades containing infectious virus particles.

The genome of BNYVV consists of four to five positive-sense single-stranded RNAs with a capped 5'-end and a 3' poly(A) tail (Gilmer et al., 2017). The replicase protein P237 is encoded by the single open reading frame (ORF) located on RNA1 (Bouzoubaa et al., 1987). The first ORF on RNA2 encodes the coat protein (CP), which is terminated by a leaky UAG stop codon allowing the translation of a readthrough protein (CP-RT) involved in vector transmission (Niesbach-Klösgen et al., 1990; Haeberlé et al., 1994; Tamada et al., 1996). The next three ORFs on RNA2 encode the triple gene block proteins 1–3 that are responsible for cell-to-cell movement (Gilmer et al., 1992). The last ORF on RNA2 encodes the viral suppressor of gene silencing (Chiba et al., 2013). The pathogenicity factor P25 being responsible for the severe rhizomania disease symptoms in sugar beet is encoded by RNA3 (Tamada et al., 1999). One ORF can be found on RNA4 encoding P31 that is required for successful vector transmission (Tamada and Abe, 1989). Certain isolates (P-and J-type) carry an additional RNA5 encoding P26.

The successful control of rhizomania disease relies only on the cultivation of resistant varieties preventing high yield losses. The Holly Sugar Company identified in 1983 the first resistance gene (*Rz1*) effective against BNYVV (Lewellen et al., 1987). It is a single dominant gene that confers partial resistance reducing virus multiplication and preventing symptom development (Lewellen et al., 1987; Scholten et al., 1996). However, the usage of *Rz1* as single resistance source over many years caused a high selection pressure on the viral population leading to the development of resistance-breaking virus variants in sugar beet growing areas worldwide (Liu et al., 2005; Pferdmenges et al., 2009; Bornemann et al., 2014; Yilmaz et al., 2018). Sequence analysis revealed a high variability within a stretch of four amino acids at position 67–70 (tetrad) located in P25 (Schirmer et al., 2005; Liu and Lewellen, 2007; Acosta-Leal et al., 2008; Acosta-Leal et al., 2010; Chiba et al., 2011). Frequent tetrad variants associated with resistance-breaking are AYPR, VCHG, and VLHG (Liu and Lewellen, 2007; Bornemann et al., 2014). Recently, Galein et al. (2018) reported the presence of 21 variants within a small sugar beet growing area located in the Pithivier region (France). The association of different mutations in the tetrad with resistance-breaking is based solely on field or greenhouse bait plant test using natural virus populations. Natural BNYVV populations display a high sequence variability in P25 (Schirmer et al., 2005) and a single population can harbor several tetrad variants with resistance-breaking and non-resistance-breaking abilities (Bornemann and Varrelmann, 2013). Therefore, only the application of a reverse genetic system allows a clear identification of resistance-breaking tetrad variants. However, there is only one study in which an isolate containing RNA1+2, supplemented with an RNA3 infectious clone was applied to confirm that a single mutation of amino acid 67 from alanine to valine promotes increased virus accumulation in *Rz1* resistant plants (Koenig et al., 2009a).

A highly variable tetrad is mainly found in A-type isolates rather than in B- or P-type isolates. However, P-type isolates are also able to overcome *Rz1* resistance (Bornemann and Varrelmann, 2013), but the tetrad in P25 contains a variant (SYHG) that is highly conserved across different isolates and has not been detected in A- or B-type isolates yet (Schirmer et al., 2005; Chiba et al., 2011; Galein et al., 2018). Furthermore, it is reported that the presence of RNA5 in P-type isolates induces a severe rhizomania phenotype (Heijbroek et al., 1999). This suggests that *Rz1* resistance-breaking by the P-type might depend on RNA5 rather than on P25 tetrad mutations. Interestingly, Koenig et al. (2009b) found in field root samples of *Rz1* resistant plants from the Pithiviers area genome reassortments consisting of either A-/P-type or B-/P-type. More recently, Galein et al. (2018) confirmed this observation in the same area and could even identify plants containing A-, B-, and P-type. Both studies indicate genome reassortments between different BNYVV types, but it remains to be shown whether the presence of P-type RNA5 mediates *Rz1* resistance-breaking in the background of A- or B-type.

Although *Rz1* has been overcome by BNYVV in certain areas, it remains the most important resistance source because of its widespread use. Thus, molecular tools are strongly required to study the effect of single mutations and genome components on *Rz1* resistance-breaking. Recently, we developed an infectious complementary DNA (cDNA) clone of BNYVV A-type, which was applied in the present study to gain insight into the mechanisms of *Rz1* resistance-breaking in sugar beet. The infection with a clonal BNYVV population is mediated by mechanical inoculation excluding the effect of the vector and other biotic factors on resistance-breaking. We identified independent mechanisms allowing BNYVV to overcome *Rz1* resistance. Moreover, we used a fluorescent-labeled clone of BNYVV to track the virus in a susceptible and resistant genotype. Our results demonstrate the feasibility of the reverse genetic system for resistance-breaking analysis and illustrate the genome plasticity of BNYVV allowing the virus to adapt rapidly to plant resistance traits.

## Material and Methods

### Plant Material and Virus Inoculation

A susceptible (KWS03) and a *Rz1* resistant genotype (BETA4430) provided by KWS Saat SE (Germany, Einbeck) was used for all infection experiments. In all experiments, young sugar beet seedlings of both genotypes were mechanically inoculated with BNYVV using the protocol from Bornemann and Varrelmann (2011) with slight modifications. To produce the inoculum for mechanical inoculation of sugar beets, plants of *Beta macrocarpa* were agroinfiltrated with the BNYVV infectious cDNA clone when the first two leaves (BBCH12) were fully developed. One true leaf and both cotyledons were inoculated with *Rhizobium radiobacter* (syn. *Agrobacterium tumefaciens/Agrobacterium fabrum*) strain GV2260 according to Voinnet et al. (1998) with an OD600 of 0.5. The inoculum consisted of BNYVV RNA1-3 cDNA clones mixed in equal amounts (Laufer et al., 2018b). Plants were kept for symptom development in a climate chamber at day and night temperatures of 24 and 18°C, respectively, and a 14 h photoperiod. Leaves displaying systemic symptoms were harvested (1 g) and grinded in 4 ml 0.05 M phosphate buffer [1 g of KH_2_PO_4_ and 7.2 g of Na_2_HPO_4_ in 1 L of diethyl pyrocarbonate (DEPC)-treated water, pH 7 to 7.4]. For inoculation of sugar beets, the plant sap obtained from infected *B*. *macrocarpa* leaves was added to a 15 ml conical centrifuge tube (Sarstedt AG & Co. KG) containing six sugar beet seedlings (7-day old) and 40 mg of carborundum (Sigma Aldrich Chemie GmbH). The tube was then mixed on a Vortex Genie 2 (Scientific Industries) at maximum speed for 50 s, and then upside down for additional 10 s to ensure inoculation of cotyledons. Plants were left for an additional 5 min in the inoculum suspension. Seedlings were washed in tap water and planted in sterile soil covered for two days with plastic bags. Each treatment consisted of 12 plants grown in a climate chamber after inoculation as described above. A double antibody sandwich enzyme-linked immunosorbent assay (DAS-ELISA) was applied to measure the virus content in lateral roots (100–150 mg) of sugar beets. Antibodies specific for BNYVV CP (AS-0737) were obtained from the Leibniz Institute DSMZ-German Collection of Microorganisms and Cell Cultures (Germany, Braunschweig). The root material was grinded in sample buffer for 45 s at 5,000 rpm using the Precellys 24 tissue homogenizer (Bertin Instruments). The ELISA was conducted according to the manufacturer's instructions. Raw absorbance values measured at 405 nm were corrected by subtraction of blank and buffer control. Only samples with an absorbance value above 0.1 were considered as positive.

In order to study the leaf phenotype of the susceptible and resistant genotype in response to BNYVV infection, several fully developed leaves from 8 to 10 weeks old sugar beet plants were mechanically inoculated with plant sap produced from *B*. *macrocarpa* as described above. The plant sap was spread onto the leaves with a cotton swab and carborundum. Inoculated plants were kept for symptom development in a climate chamber at day and night temperatures of 24 and 18°C, respectively, and a 14 h photoperiod.

### Construction of Infectious RNA3 and RNA5 Clones

A wild type RNA3 containing the tetrad composition AYPR was cloned from a *Rz1* resistance-breaking isolate (A type) obtained from the Netherlands (Bornemann et al., 2014). The RNA5 clone was derived from a natural P-type population collected in Pithivier. For virus isolation, bait plants were grown in infested soil samples as described by Bornemann et al. (2014). Lateral roots (100–150 mg) were harvested and subjected to RNA extraction using the NucleoSpin RNA Plant kit (Macherey-Nagel). The sample material was grinded in extraction buffer for 45 s at 5,000 rpm using the Precellys 24 tissue homogenizer. All subsequent steps were conducted according to the manufacturer's instructions. Reverse transcription of RNA (500 ng) was done with the RevertAid H Minus reverse transcriptase and oligo(dT)18 primer (Thermo Fisher). The full-length RNA3 and RNA5 was amplified with specific primers (**Supplementary Table 1**) using the Phusion Flash High-Fidelity PCR Master Mix (Thermo Fisher). PCR products were gel purified using the NucleoSpin Gel and PCR Clean-up system according to the manufactures instructions (Macherey-Nagel). Gibson assembly was applied as *in vitro* recombination method for the cloning of full-length cDNAs under control of 35S promoter from cauliflower mosaic virus into the linearized plasmid pDIVA according to Laufer et al. (2018b). After assembly, *in vitro* recombination products were transformed into chemical competent *Escherichia coli* cells (strain DH5α) as described by Inoue et al. (1990). Plasmids carrying the RNA3 (EcoRI/XbaI: 3,544 bp, 2,561 bp) or RNA5 (EcoRI/XbaI: 3,324 bp, 2,137 bp, 220 bp) were identified by means of appropriate fast restriction enzyme digest (Thermo Fisher) and verified by commercial capillary Sanger sequencing (Eurofins MWG Operon). Subsequently cDNA clones were transformed into *R. radiobacter* strain GV2260 (Voinnet et al., 1998). Full-length sequences of RNA3 (MN148886) and RNA5 (MN148887) were deposited in the databases of the National Center for Biotechnology Information (NCBI).

### Site-Directed Mutagenesis of P25 Open Reading Frame

Site-directed mutagenesis of P25 was achieved by amplification of the plasmid pDIVA containing the full-length BNYVV RNA3 cDNA clone with specific mutated primers, followed by re-ligation. Specific primers introducing mutated nucleotides are listed in **Supplementary Table 1**. All PCRs were carried out with the Phusion Flash High-Fidelity PCR Master Mix. PCR products were gel purified as described above. Subsequently, the PCR amplification products were phosphorylated by further reaction with T4 Polynucleotide Kinase (Promega) and ligated with T4 DNA Ligase (Promega) or directly used for transformation. Plasmids were transformed into chemical competent *E. coli* cells (strain DH5α) as described above. Plasmids carrying the correct insert (SacI/EcoRI: 3,524 bp, 2,346 bp, 236 bp) were identified by means of appropriate fast restriction enzyme digest and all introduced mutations were confirmed by commercial capillary Sanger sequencing. Subsequently cDNA clones were transformed into *R. radiobacter* strain GV2260 as described above.

### Next Generation Sequencing

To study the selection pressure on P25 exerted by *Rz1* resistance, the sequence variability of P25 was determined by next generation sequencing (NGS) after one host passage in four plants from the susceptible and resistant genotype. Total RNA extraction from lateral roots of infected plants and reverse transcription was done as described above. The amplicon library was prepared by two successive PCRs according to Liebe et al. (2016). In brief, the nearly complete P25 ORF (RNA3 nt 102-601) was initially amplified with primers P25-seq-fw/P25-seq-rv (**Supplementary Table 1**) using the Phusion Flash High-Fidelity PCR Master Mix. Both primers contain an adaptor with specific sequences necessary for NGS. PCR cycle conditions were as follows: initial DNA denaturation step of 98°C for 10 s followed by 30 cycles of 98°C for 5 s, primer annealing at 60°C for 5 s, 72°C for 15 s, and a final elongation at 72°C for 5 min. Bands of the correct size were gel excised and purified as described above. One microliter of purified product was amplified in a second PCR to add a sample specific index for barcoding using D501fw as forward primer and D701rv–D709rv as reverse primers (**Supplementary Table 1**). The primer D501fw was always used as forward primer whereas the primers D701rv–D709rv were sample specific. PCR cycle conditions were as follows: initial DNA denaturation step of 98°C for 10 s followed by 20 cycles of 98°C for 5 s, 44°C for 5 s, 72°C for 15 s, and a final elongation at 72°C for 5 min. To exclude artificial mutations generated during PCR or NGS, P25 amplified from the RNA3 plasmid used for inoculation was used as internal control. All PCR products were again gel purified and analyzed on a Bioanalyzer using 1000 Chip (Agilent, CA, USA). DNA concentrations were measured by means of the QuantiT™ PicoGreen DNA Assay Kit (Invitrogen, CA, USA) on an Infinite Reader M200 instrument (Tecan, Switzerland). For each amplicon library preparation, 100 ng DNA were processed applying the TruSeq Nano DNA Sample Preparation Kit (Illumina, CA, USA) included sequencing adapter ligation according to the protocol provided by the manufacturer. Resulting quantified sequencing libraries were sequenced on a MiSeq System (Illumina) in paired-end mode (2 x 300 bp sequencing cycles) using the MiSeq Reagent Kit v3 (Illumina) following manufacturer's instructions. Raw sequencing data are available in the European Nucleotide Archive (EBI) under the project accession number PRJEB34221. After sequencing, raw data were imported into an in-house processing pipeline (Liebe et al., 2016) for sequencing primer and barcode trimming. The processed sequencing reads were mapped using bowtie 2 (v2.1.0) onto P25 (Langmead and Salzberg, 2012). Mapped sequence reads were analyzed for single-nucleotide polymorphisms (SNPs) in the ReadXplorer platform 2.2.3. SNPs with more than 10% frequency were included in the analysis (Hilker et al, 2014).

### Confocal Laser Scanning Microscopy

To study the viral distribution within root and leaf tissue of the susceptible and resistant genotype, plants were infected with the mRFP fluorescently-labeled full-length clone of RNA2 (Laufer et al., 2018a) as described above. The mRFP fluorescence was visualized with the TCS-SP5 confocal laser-scanning microscope (Leica Microsystems). Excitation/emission wavelengths for mRFP was 561 nm/520–540 nm. All confocal images were processed with the LAS-AF software version 2.6.3.8173 (Leica Microsystems).

### Statistical Analysis

Statistical analysis of ELISA absorbance values was carried out with SAS Version 9.3 (SAS Institute Inc., Cary, USA). Only plants considered as infected were included in the analysis, except for treatments without any infected plant. In this case, absorbance values from all plants were used for statistical analysis. The MIXED procedure in SAS was used for analysis of variance (ANOVA) which accounts for unequal sample size between treatments. Residuals were tested for normality using the Kolmogorov-Smirnov test (P < 0.05), and if necessary, data were transformed to approximate normal distribution. Variance heterogeneity was considered by estimating different covariance parameter for each effect using the “GROUP = “ syntax in SAS. Based on the Akaike Information Criterion (AIC), the best-fit model was selected and tested against the null model (one covariance) using the likelihood ratio test implemented in the MIXED procedure (P ≤ 0.05). All factors were considered as fixed effects and tested for significance in the F statistic (P ≤ 0.05). The denominator degrees of freedom for the tests of fixed effects were approximated according to the method of Kenward and Roger (1997). When factors or interactions were significant (P < 0.05), means were separated by the Tukey Kramer test (P < 0.05) using the macro PDMIX800 (Saxton, 1998). The R package ggplot2 was used for data visualization (Wickham, 2009).

## Results

### *Rz1* Mediates a Root Specific Resistance Toward Beet Necrotic Yellow Vein Virus

To allow studying *Rz1* resistance-breaking with a BNYVV reverse genetic system, a clear discrimination between susceptible and resistant genotypes is required. Therefore, we analyzed in experiment 1 the virus content in lateral roots of both genotypes after infection with the non-resistance-breaking BNYVV clone containing the P25 tetrad motif ALHG (**Table 1**, experiment 1). The susceptible genotype accumulated considerable amounts of BNYVV with 9 infected out of 12 inoculated plants. Furthermore, characteristic leaf symptoms including vein yellowing and necrosis indicative for systemic infection were observed (**Supplementary Figure S1**). In contrast, the *Rz1* resistant genotype displayed an ELISA value comparable to the healthy control and statistically different from the susceptible genotype. Moreover, all plants from the resistant genotype displayed a similar ELISA value and were considered as non-infected (**Supplementary Figure S2**). However, we could observe systemic leaf symptoms indicated by small yellow lesions (**Supplementary Figure S1**). These leaves were not developed when plants had been inoculated indicating that BNYVV has moved into newly developing leaves. Furthermore, the symptoms in *Rz1* plants induced by the non-resistance-breaking clone were observed in all subsequent experiments although the ELISA values measured in roots were comparable to the healthy control. Therefore, we assumed that the *Rz1* resistance mechanism prevents infection in the taproot but not in leaves. This prompted us to compare the leaf phenotype of the susceptible and resistant genotype after mechanical inoculation of the non-resistance-breaking BNYVV clone. Plant sap produced from systemically infected *B*. *macrocarpa* leaves was used as inoculum for sugar beet. After inoculation, both genotypes developed yellow local lesions that became later necrotic within the center (**Supplementary Figure S3**). Neither the symptom onset, nor lesion shape was different between the genotypes. This indicated a root specific *Rz1* resistance toward BNYVV in the taproot that is different from the local lesion resistance response observed on leaves.

**Table 1:** Effect of different mutations in P25 on the average ELISA absorbance value (A405) measured in lateral roots of sugar beets. The experiments 1–3 were conducted in a susceptible and *Rz1* resistant sugar beet cultivar with an infectious clone of beet necrotic yellow vein virus (BNYVV) A-type. Either mutations were introduced directly into the open reading frame (ORF) from P25 or the complete RNA3 was exchanged to study the effect on resistance-breaking. The wild type RNA3s with the tetrad variant ALHG and AYPR are indicated by wt. Statistically significant differences between treatments are indicated by small letters (p values < 0.5).

Experiment and virus variant		Susceptible genotype			*Rz1* resistant genotype		
		Infected plants/inoculated plants^a^	Plants with systemic symptoms	Mean A405 of infected plants^b^	Infected plants/inoculated plants^a^	Plants with systemic symptoms	Mean A405 of infected plants^b^
**Experiment 1 (52 dpi)**						
BNYVV ALHG wt		9/12	6	0.793 (0.221) *a*	0/12	3	0.009 (0.025) *b*
Healthy		0/12	0	−0.005 (0.002) *b*	0/12	0	−0.001 (0.005) *b*
**Experiment 2 (49 dpi)**						
BNYVV ALHG wt		9/12	4	0.762 (0.522) *ab*	0/12	5	0.016 (0.022) *de*
BNYVV ALHG → AYPR		10/12	7	0.548 (0.212) *abc*	8/12	8	0.351 (0.117) *bcd*
BNYVV ALHG → VCHG		9/12	5	0.472 (0.224) *abc*	4/12	5	0.189 (0.150) *cde*
BNYVV ALHG → VLHG		10/12	6	0.611 (0.243) *abc*	5/12	5	0.215 (0.098) *cde*
BNYVV AYPR wt		8/12	7	0.823 (0.303) *a*	10/12	8	0.549 (0.188) *abc*
Healthy		0/12	0	0.011 (0.007) *e*	0/12	0	−0.013 (0.027) *e*
**Experiment 3 (44 dpi)**						
BNYVV AYPR wt		12/12	8	0.602 (0.314) *b*	12/12	6	0.614 (0.281) *b*
BNYVV AYPR P25-S42L		8/12	5	0.676 (0.120)*b*	8/12	6	0.655 (0.335) *b*
BNYVV AYPR → ALHG		11/12	10	1.063 (0.243) *a*	0/12	5	0.015 (0.002)*c*
Healthy		0/12	0	−0.008 (0.005) *c*	0/12	0	−0.010 (0.006) *c*

^a^Only samples with an ELISA absorbance value above 0.1 were considered as positive.

^b^Mean A405 was calculated using only ELISA absorbance values obtained from infected plants, except for treatments without any infected plant. In this case, absorbance values from all plants were used for mean calculation. Numbers in brackets indicate standard deviation.

### Different Mutations of P25 Amino Acids 67–70 Promote Increased Virus Accumulation in *Rz1* Genotype

Since the non-resistance breaking clone was unable to infect plants from the resistant genotype as described above, any increase in the virus concentration would indicate a resistance-breaking event. To provide experimental evidence for the involvement of the P25 tetrad in *Rz1* resistance-breaking, we replaced in the non-resistance-breaking BNYVV clone the tetrad motif ALHG by the motifs AYPR, VCHG, and VLHG, respectively, which are associated with resistance-breaking. Furthermore, we cloned a RNA3 cDNA from a natural A-type *Rz1* resistance-breaking virus population harboring the tetrad motif AYPR. All tetrad variants induced rhizomania like leaf symptoms in sugar beets confirming systemic movement of BNYVV (**Supplementary Figure S4**). As described above, the motif ALHG present in the wild type BNYVV clone allowed no virus accumulation in lateral roots of the resistant genotype (**Table 1**, experiment 2). In contrast, the replacement of ALHG by AYPR, VCHG, and VLHG promoted increased virus accumulation in the resistant genotype. The presence of each mutated tetrad was confirmed by conventional Sanger sequencing at the end of the experiment in individual plants. However, these variants were statistically not different from the ALHG clone, although ELISA values from single plants indicated an obvious effect on virus accumulation in the resistant genotype (**Supplementary Figure S2**). This is probably due the large standard deviation and unequal sample size resulting in a low power of the statistical test. The wild type RNA3 from the resistance-breaking virus population also enabled virus accumulation in the resistant genotype, and this effect was statistically different from the wild type RNA3 ALHG. Interestingly, the absorbance values measured in the resistant genotype were somewhat higher compared to those induced by the BNYVV clone in which ALHG was replaced by AYPR. Therefore, we checked the sequence of P25 from the wild type RNA3 for the presence of mutations outside the tetrad motif AYPR. An amino acid substitution from leucine to serine could be identified at position 42, which has been also found in other BNYVV virus populations carrying the AYPR motif (Fernando Gil et al., 2018). To test the effect on resistance-breaking, we replaced amino acid 42 (serine) by leucine (S42L) which is present in our non-resistance-breaking BNYVV clone. However, virus accumulation measured in the susceptible and resistant genotype revealed no differences compared to the wild type RNA3 (**Table 1**, experiment 3). Furthermore, we mutated the tetrad motif AYPR in the wild type RNA3 to ALHG, which completely abolished virus accumulation in the resistant genotype similar to our non-resistance-breaking BNYVV clone. Moreover, it resulted in a significant increase in the virus concentration of the susceptible genotype supporting an effect of the tetrad on the virus fitness.

### Virus Distribution of Beet Necrotic Yellow Vein Virus in the Susceptible and Resistant Sugar Beet Genotype

**Figure 1:**
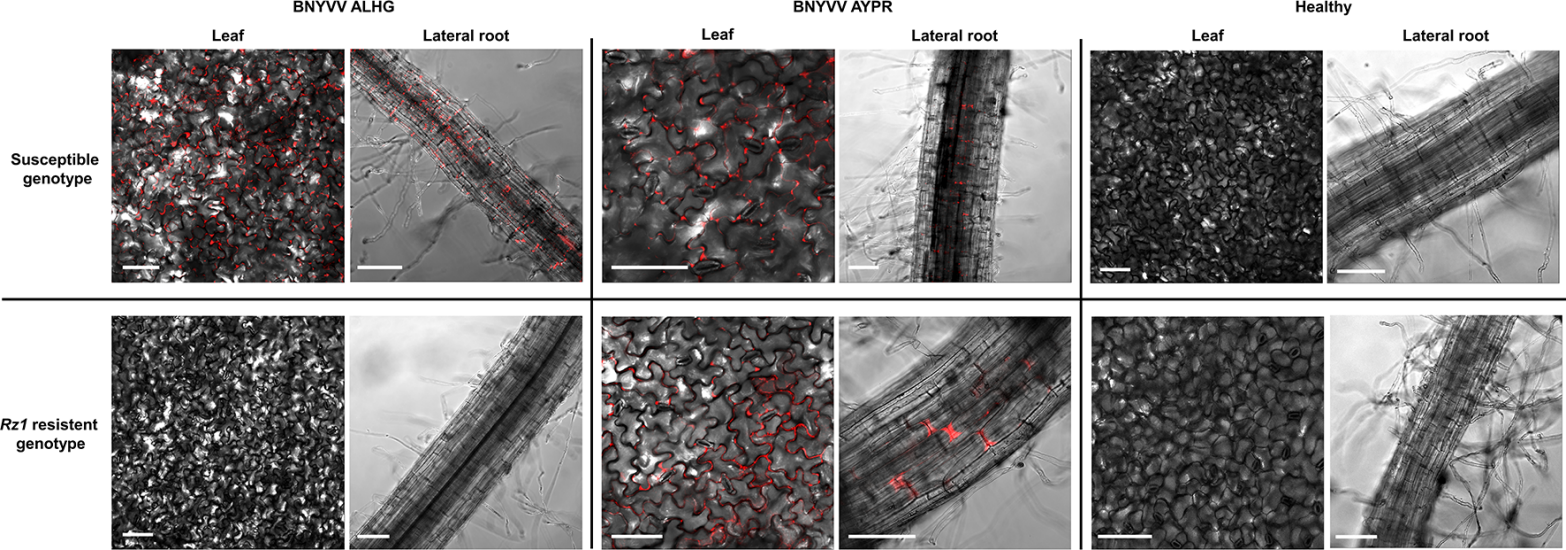
Confocal imaging of sugar beet leaf tissue and lateral roots (30–50 dpi) systemically infected with the fluorescently complementary DNA (cDNA) clone of beet necrotic yellow vein virus (BNYVV) compared to the healthy control. Plants were infected with a non-resistance-breaking RNA3 clone (ALHG) and with a RNA3 cloned from a resistance-breaking virus population (AYPR). White bars indicate the selected scale of 50 µm.

The results from the above described experiments suggested that the *Rz1* based resistance response reduces virus replication below the ELISA detection limit since plants infected with the non-resistance-breaking BNYVV clone were comparable to the healthy control. To study the virus replication of BNYVV in infected plants of both genotypes, we used a recently developed fluorescence labeled cDNA clone of BNYVV (Laufer et al., 2018a). In the RNA2 cDNA clone, the CP-RT had been substituted by an mRFP ORF. The virus replication was tested with the RNA3 carrying the tetrad motif ALHG and with the wild type RNA3 from the resistance-breaking virus population (AYPR). With this approach, it was our aim to prove whether the absence or presence of virus-expressed fluorescence in lateral roots correlates with the resistance-breaking ability of BNYVV. In case of the susceptible genotype we could observe in lateral roots and leaves of infected plants a clear fluorescence signal indicating virus replication in both plant organs (**Figure 1**). Fluorescence in leaves was limited to tissue displaying systemic symptoms. Lateral roots and leaves of the resistant genotype only displayed fluorescence, if plants were inoculated with the cDNA clone containing RNA3 with the AYPR tetrad motif. We never observed any fluorescence in *Rz1* resistant plants infected with the non-resistance-breaking BNYVV clone. However, these plants developed systemic leaf symptoms, but no fluorescence was observed in this tissue suggesting a deletion of the mRFP ORF. Therefore, we checked by PCR the presence of the mRFP ORF in infected leaf tissue using flanking primers. Only smaller fragments of the mRFP ORF were amplified from the resistant genotype whereas the full product was obtained from the susceptible genotype (**Supplementary Figure S5**). Interestingly, virus fluorescence could be still observed in primarily inoculated cotyledons (data not shown) indicating that the mRFP ORF was partially deleted by recombination after inoculation of the non-resistance-breaking BNYVV clone into the resistant genotype.

### Spontaneous Deletion of Amino Acid 179 in P25 Promotes Virus Accumulation in the *Rz1* Sugar Beet Genotype

In the field under natural growth conditions, it took only a few sugar beet cultures in a 2–3 year rotation to select *Rz1* resistance-breaking strains. We aimed to find out, if *Rz1* resistant-breaking mutations occur already after one host passage of 6 weeks in resistant plants. To study the effect of the selection pressure on P25 ORF, the sequence variability was determined by NGS after one host passage of the ALHG containing recombinant BNYVV in the susceptible and resistant genotype. Plants from the resistant genotype selected for this experiment displayed a virus content below the threshold. However, it was possible to prepare a P25 amplicon library for NGS. The internal control comprising P25 amplified from the RNA3 plasmid was free of sequence errors after quality filtering. In total, we identified 10 nucleotide mutations of which six were non-synonymous (**Table 2**). The mutation of amino acid 117 from serine to phenylalanine (S117F) in P25 was only identified in the resistant genotype. The amino acid 140 was mutated from valine to glycine (V140G) and was found in plants from both genotypes. The deletion of three consecutive nucleotides determined in one infected plant of the resistant genotype caused a deletion of amino acid 179 (Δ179). Remarkably, no sample displayed a mutation of the tetrad at amino acid positions 67–70. We further counted the number of reads for each nucleotide mutation to describe the frequency within the virus population (**Table 2**). For S117F and V140G, reads carrying the nucleotide from the reference sequence were overrepresented and only a small number of reads harbored a mutated nucleotide leading to the amino acid exchange. Interestingly, reads with mutations at nucleotide positions 534–536, leading to the deletion of amino acid 179, were overrepresented in the population. We subsequently introduced all non-synonymous mutations into the non-resistance-breaking BNYVV clone and tested their resistance-breaking ability (**Table 3**, experiment 4). The infectivity of BNYVV in the susceptible genotype was retained independent of the mutation, but a considerable fitness penalty was observed particularly for V140G. This mutation also abolished development of systemic symptoms in plants from *B*. *macrocarpa* (**Supplementary Figure S6**) and sugar beet (**Supplementary Figure S7**). The deletion of amino acid 179 altered symptom expression in *B*. *macrocarpa*, but this effect was less pronounced in sugar beet. No effect on symptom development was observed for mutation S117F. No virus content was measured in plants of the resistant genotype except for one mutation. The mutation Δ179 led to a significant higher virus concentration in the *Rz1* genotype confirming the resistance-breaking ability. Interestingly, a mutation of amino acid 179 from asparagine to aspartic acid (N179D) had been observed in a natural BNYVV population (Chiba et al., 2008), but an effect on *Rz1* resistance-breaking has not been proven yet. Therefore, we introduced this mutation into the non-resistance-breaking BNYVV clone and compared it with the mutation Δ179 (**Table 3**, experiment 5). The mutation N179D had no effect on resistance-breaking and only Δ179 promoted virus accumulation in the resistant genotype. Finally, leaves of the susceptible and resistant genotype were rub-inoculated with plant sap obtained from *B*. *macrocarpa* leaves infected with BNYVV carrying the mutation Δ179 (**Supplementary Figure S8**). No differences were observed between both genotypes and the local lesions induced by BNYVV carrying the mutation Δ179 were comparable to the wild type (**Supplementary Figure S3**). This indicates that *Rz1* resistance-breaking in lateral roots mediated by the mutation Δ179 has no effect on the phenotype resistant response in leaves.

**Table 2:** Non-synonymous substitutions in P25 open reading frame (ORF) identified by deep sequencing of a clonal beet necrotic yellow vein virus (BNYVV) population from a susceptible and resistant genotype. Nucleotide mutations leading to amino acid exchanges were analyzed in four plants from the resistance (1–4) and susceptible (5–8) genotype, respectively. The number of reads containing the mutated (underlined) or the reference nucleotides (bold) were counted for each mutation.

Genotype	Plant	Nucleotide position	Reference nucleotide*	Mutated nucleotide	Reads A	Reads C	Reads G	Reads T	Deletion	Amino acid exchange
Resistant	4	350	C	T	10	**183,041**	12	23,703	18	S117F (serine → phenylalanine)
Susceptible	8	419	T	G	41	77	844	**171,896**	2	V140G (valine → glycine)
Resistant	2	419	T	G	68	49	1,020	**215,308**	2	V140G (valine → glycine)
Resistant	3	534	T	–	0	1	7	**1,914**	210,256	Δ179 (deletion of asparagine)
Resistant	3	535	A	–	**1,902**	2	37	13	210,224	
Resistant	3	536	A	–	**1,774**	49	194	95	210,066	

*The reference sequence of the BNYVV RNA3 clone is deposited in the databases of the National Center for Biotechnology Information (Accession number: KX665538).

**Table 3:** Effect of different mutations in P25 on the average ELISA absorbance value (A405) measured in lateral roots of sugar beets. The experiments 4 and 5 were conducted in a susceptible and Rz1 resistant sugar beet cultivar with an infectious clone of beet necrotic yellow vein virus (BNYVV) A-type. Mutations were introduced into the open reading frame (ORF) from P25 to study the effect on resistance-breaking. The wild type RNA3 with the tetrad variant ALHG is indicated by wt. Statistically significant differences between treatments are indicated by small letters (p values < 0.5).

Experiment and virus variant	Susceptible genotype			*Rz*1 resistant genotype		
	Infected plants/inoculated plants^a^	Plants with systemic symptoms	Mean A405 of infected plants^b^	Infected plants/inoculated plants^a^	Plants with systemic symptoms	Mean A405 of infected plants^b^
**Experiment 4 (41 dpi)**						
BNYVV ALHG P25-S117F	8/12	1	0.678 (0.374) *ab*	0/12	1	−0.002 (0.040) *de*
BNYVV ALHG P25-V140G	7/12	0	0.167 (0.069) *cd*	0/12	0	0.022 (0.059) *e*
BNYVV ALHG P25-Δ179	6/12	2	0.393 (0.279) *bc*	9/12	5	1.009 (0.201) *a*
Healthy	0/12	0	−0.025 (0.004) *e*	0/12	0	−0.020 (0.008) *e*
**Experiment 5 (43 dpi)**						
BNYVV ALHG wt	7/12	6	0.831 (0.388) *a*	0/12	11	0.026 (0.024) *b*
BNYVV ALHG P25-N179D	10/12	0	0.805 (0.369) *a*	0/12	5	0.014 (0.014) *b*
BNYVV ALHG P25-Δ179	7/12	4	0.694 (0.327) *a*	7/12	5	0.797 (0.143) *a*
Healthy	0/12	0	0.026 (0.002) *b*	0/12	0	0 (0.005) *b*

^a^Only samples with an ELISA absorbance value above 0.1 were considered as positive.

^b^Mean A405 was calculated using only ELISA absorbance values obtained from infected plants, except for treatments without any infected plant. In this case, absorbance values from all plants were used for mean calculation. Numbers in brackets indicate standard deviation.

### BNYVV RNA5 Promotes Increased Virus Accumulation in the *Rz1* Genotype

We cloned a RNA5 from a P-type population that had been previously demonstrated to overcome *Rz1* resistance (data not shown) to study its effect on *Rz1* resistance stability. The RNA5 cDNA clone was added to the non-resistance-breaking BNYVV clone consisting of RNA1-3 (**Table 4**, experiment 6). The addition of RNA5 had no effect on the virus content measured in the susceptible genotype, but it promoted increased absorbance values in the resistant genotype compared to the healthy control. This effect was statistically not significant due to the above mentioned reasons, but ELISA values from individual plants clearly support virus accumulation in the resistant genotype (**Supplementary Figure S2**). However, the virus accumulation was still inhibited indicated by relatively low ELISA values. This prompted us to skip the RNA3 from the inoculum as it might be targeted by the resistance mechanism. The absence of RNA3 significantly lowered the virus concentration in the susceptible genotype and had no effect on the virus content in the resistance genotype. Nevertheless, virus accumulation in the absence of RNA3 demonstrated that RNA5 can complement the function of RNA3 in systemic movement. In sugar beet, no additional effect of RNA5 on symptom development was observed, if RNA3 was present (**Supplementary Figure S9**). In the absence of RNA3, no systemic symptoms were observed in sugar beet, but faint systemic symptoms induced by RNA5 could be observed in *B*. *macrocarpa* (**Supplementary Figure S10**).

**Table 4:** Effect of beet necrotic yellow vein virus (BNYVV) RNA5 on the average ELISA absorbance values (A405) measured in lateral roots of sugar beets. The experiment 6 was conducted in a susceptible and Rz1 resistant sugar beet cultivar with an infectious clone of BNYVV A-type. The wild type RNA3 with the tetrad variant ALHG is indicated by wt. The presence of RNA5 in the inoculum is indicated by + whereas the absence of RNA3 is indicated by −. Statistically significant differences between treatments are indicated by small letters (p values < 0.5).

Experiment and virus variant	Susceptible genotype			*Rz*1 resistant genotype		
	Infected plants/inoculated plants^a^	Plants with systemic symptoms	Mean A405 of infected plants^b^	Infected plants/inoculated plants^a^	Plants with systemic symptoms	Mean A405 of infected plants^b^
**Experiment 6 (47 dpi)**						
BNYVV ALHG wt	12/12	11	0.724 (0.016) *a*	0/12	3	0.005 (0.003) *cd*
BNYVV ALHG + RNA5	12/12	10	0.660 (0.358) *a*	8/12	10	0.213 (0.112) *bcd*
BNYVV − RNA3 + RNA5	12/12	0	0.416 (0.165) *b*	8/12	0	0.255 (0.100) *bc*
Healthy	0/12	0	0.001 (0.001) *d*	0/12	0	0.001 (0.001) *d*

^a^Only samples with an ELISA absorbance value above 0.1 were considered as positive.

^b^Mean A405 was calculated using only ELISA absorbance values obtained from infected plants, except for treatments without any infected plant. In this case, absorbance values from all plants were used for mean calculation. Numbers in brackets indicate standard deviation.

## Discussion

Plant viruses possess a highly flexible genome allowing virus populations a rapid adaption to their host plant. A high selection pressure conferred by single resistance genes favor the development of resistance-breaking mutations that become dominant in the virus population. Several resistance-breaking events have been reported in the past for important host-virus-systems like barley-barley yellow mosaic virus (Li et al., 2016), rice-rice yellow mottle virus (Pinel-Galzi et al., 2016), and tomato-tomato spotted wilt virus (Batuman et al., 2017). Therefore, a continuous observation of virus populations is required in order to identify new mutations, which need to be tested subsequently for their resistance-breaking ability. Our vector-free reverse genetic system allowed a clear discrimination between susceptible and *Rz1* resistant sugar beet genotypes following infection with a non-resistance-breaking BNYVV clone. The virus content in the resistant genotype was always comparable to the healthy control demonstrating that the resistance mechanism effectively blocked virus replication. In previous studies applying natural virus populations and vector transmission by *P*. *betae*, the *Rz1* mediated resistance response resulted in a reduction of the virus content but never below the detection limit (Liu and Lewellen, 2007; Bornemann et al., 2014). This discrepancy between our and previous studies might be due to differences in the infection method. Although it is little known about the *Rz1* resistance mechanism, it is unambiguous that the initial infection of lateral roots by *P*. *betae* is not prevented, but the subsequent virus replication and movement is dramatically reduced. Whether this can result in detectable amounts of BNYVV virions is unknown. Thus, it is also possible that the virus content within cytosori and zoospores of *P*. *betae* is measured, leading to elevated ELISA values in resistant cultivars.

In contrast to root infection, the leaf phenotype obtained after mechanical inoculation of BNYVV allowed no differentiation between the susceptible and resistant genotype. Therefore, our results are in line with previous observations (Chiba et al., 2008) and provide a clear evidence for a root specific *Rz1* resistance response toward BNYVV that must be different from the response of the leaf tissue. This is also supported by the observation of systemic symptoms in the resistant genotype after inoculation of the non-resistance-breaking BNYVV clone. Although this is due to an artifact produced by the mechanical inoculation with sap, it shows the ability of BNYVV to replicate in leaves of the resistant genotype despite a clear resistance response in lateral roots. Moreover, the high number of systemically infected plants observed in all experiments indicates that mechanical inoculation of whole sugar beet seedlings promotes systemic movement of BNYVV, which is rarely observed in the field.

Our results confirm the *Rz1* resistance-breaking ability of two new mutations (AYPR and VLHG) which have not been tested yet. Our findings are in line with a previous study from Koenig et al. (2009a) showing that a mutation from AHHG to VCHG enables BNYVV to overcome *Rz1* resistance. Despite the clear evidence for *Rz1* resistance-breaking by mutation of the tetrad, absorbance values remained lower compared to the susceptible genotype. This might indicate the involvement of further mutations in the viral genome in resistance-breaking. Furthermore, the variability in absorbance values between AYPR, VCHG, and VLHG suggests that the tetrad variants are associated with a different fitness advantage in the resistant genotype. This might be an explanation for the large variability of different tetrad variants found in natural virus populations (Galein et al., 2018; Weiland et al., 2019), which is also indicative for an ongoing virus evolution. The replacement of the RNA3 in the non-resistance-breaking BNYVV clone by an RNA3 derived from a resistance-breaking virus population demonstrated that two A-type populations can exchange genome components leading to infectious reassortments overcoming *Rz1* resistance. The mutation of AYPR to ALHG in the wild type RNA3 abolished virus accumulation in the resistant genotype supporting the crucial role of the tetrad in *Rz1* resistance-breaking. We further tested the influence of AS42 in P25 on resistance-breaking, but no effect was identified. However, it might be possible that this mutation has effects on the interaction of P25 with itself or with other virus and plant derived proteins, which have been not identified yet. Apart from that, we could identify several mutations in the 5' and 3' non-translated region of the wild type AYPR RNA3 (data not shown), but their effect on resistance-breaking has not been proven here.

A strong *Rz1* resistance response was observed allowing no virus accumulation in lateral roots when the non-resistance-breaking BNYVV clone was inoculated. Therefore, we wanted to prove the absence of virus replication using a fluorescence labeled clone of BNYVV. Delbianco et al. (2013) developed a BNYVV RNA5 replicon expressing GFP, but we often observed a lack of fluorescence in the experimental hosts *B*. *macrocarpa* and Nicotiana *benthamiana* due to a rapid loss of RNA5 during systemic infection (data not shown). Therefore, we recently developed a clone with an alternative marker insertion position following gene replacement strategy and labeled BNYVV by integrating the mRFP ORF into RNA2 through the replacement of the CP-RT (Laufer et al., 2018a). A similar strategy for fluorescence labeling was also used by Jiang et al. (2019) for BNYVV, but systemic movement in sugar beet has not been demonstrated. We could observe a strong fluorescence in lateral roots and leaves indicative for a systemic spread of the labeled BNYVV clone. Fluorescence in leaves was restricted to the tissue displaying vein yellowing symptoms and fluorescence was never observed outside this area. To our opinion, this supports the idea that vein yellowing and necrosis induced in sugar beet are the result of a plant defense response. This is further evidenced by the restriction of BNYVV movement to yellow local lesions after mechanical inoculation of sugar beet leaves as described above. We never observed any fluorescence when the resistant genotype was inoculated with the non-resistance-breaking BNYVV clone. Moreover, the mRFP ORF underwent deletion during the incompatible interaction. We assume that the high selection pressure on the virus population during the incompatible interaction with the resistance genotype is responsible for the rapid occurrence of mRFP deletions. This is supported by the observation of fluorescence in lateral roots of the resistance genotype when plants were infected with resistance-breaking BNYVV clone. However, we cannot exclude that mRFP deletions were already present in the inoculum although fluorescence was always observed in *B*. *macrocarpa* prior to harvest of leaf material for sugar beet infection. Moreover, we occasionally observed this deletion also in the susceptible genotype, but never as frequent as in the resistant genotype. This indicates that in principle the replacement of the CP-RT by the mRFP ORF somehow interferes with viral infectivity and movement in sugar beet, which we have never observed in *B*. *macrocarpa* and *N*. *benthamiana*.

Estimation of substitution rates in P25 revealed that 14 amino acids are under a high positive selection pressure conferred by resistant cultivars, which are grown in all sugar beet growing areas with BNYVV presence (Schirmer et al., 2005). After having demonstrated the usefulness of the cDNA clone for characterization of resistance-breaking mutations, we aimed to proof whether such mutations can be selected after one host passage in a resistant genotype. The initial inoculum produced in *B*. *macrocarpa* and used for sugar beet infection was not subjected to NGS, and therefore the final mutations identified at the end of the experiment cannot be addressed to sugar beet alone. However, the inoculum used for sugar beet infection was the same for all plants, and therefore mutations manifested already in *B*. *macrocarpa* should have been found in all analyzed plants. Since this was not the case, we assume that at least the identified resistance-breaking mutation was due to the selection in the resistant genotype. This amino acid deletion was rapidly selected and the over-representation within the virus population was a clear indicator for its fitness advantage in the resistant genotype. Interestingly, despite the dominance of the deletion within the virus population, the virus content of the plant in which this mutation was selected, remained below the detection limit. Only the infection with the cDNA clone carrying this mutation allowed us to confirm its resistance-breaking ability. The amino acid 179 is also under selection in natural virus populations with an amino acid exchange from asparagine to aspartic acid present in certain isolates (Chiba et al., 2008). However, we could show here that a deletion of amino acid 179 rather than an exchange is required to overcome *Rz1* resistance. Nevertheless, we do not know whether the deletion of amino acid 179 is accompanied by a fitness penalty, which might be a reason for its missing observation in natural virus populations. It cannot be excluded that the mechanical inoculation may lead to artificial mutations on the P25 gene that might not occur or survive under field condition. The amino acid 179 is in close proximity to the nuclear export sequence (NES) located at position 169–178 (Vetter et al., 2004). It might be that the mutation negatively affects the nucleo-cytoplasmic shuttling activity of P25. Subsequently, this might interfere with the recognition of the avirulence gene of *Rz1* leading to the activation of the resistant response. Thus, the effect of the mutation Δ179 on the subcellular localization of P25 should be proven in further experiments. Apart from that, the vector transmissibility must be confirmed by loading the mutated BNYVV clone into a *P*. *betae* population (Bornemann and Varrelmann, 2011).

Finally, we also demonstrated that the supplementation of P-type RNA5 in the background of the A-type BNYVV clone promotes virus accumulation in the resistant genotype. In principle, this result evidenced the involvement of P-type RNA5 in *Rz1*-resistance-breaking. Furthermore, it demonstrates that a genome reassortment of BNYVV A-type with RNA5 from P-type is sufficient to allow BNYVV replication in *Rz1* resistant genotypes. The increased virus replication was independent of the tetrad, which further indicates that BNYVV has developed different strategies to overcome *Rz1* resistance. However, the virus content was still considerably lower compared to the susceptible genotype. This strongly supports the involvement of further mutations in *Rz1* resistance-breaking. To provide further evidence, it remains to be shown whether the tetrad motif SYHG present in most P-type isolates is necessary for *Rz1* resistance-breaking. Apart from that, we showed for the first time that RNA5 mediate systemic movement in *B*. *macrocarpa* and sugar beet in the absence of RNA3. This is probably due to the presence of the coremin motif, which has been shown to be crucial for systemic movement mediated by RNA3 (Lauber et al., 1998; Ratti et al., 2009; Peltier et al., 2012). It might be possible that the effect of RNA5 on systemic movement is directly linked with its resistance-breaking ability. Moreover, transient expression experiments conducted in *Chenopodium quinoa* suggest that P26 encoded by RNA5 is a pathogenicity enhancer (Link et al., 2005). Here, we could not observe an increase in the virus content or symptom severity, when a P-type RNA5 was co-infected with a BNYVV A-type clone. However, an infectious clone of BNYVV P-type needs to be developed to further elucidate the particular role of RNA5 for virus pathogenicity and resistance-breaking.

To sum up, we could show with the usage of a reverse genetic system that BNYVV possess a high genome plasticity allowing a rapid adaption to plant resistance traits. Moreover, the results from NGS analysis of the P25 ORF indicate that the genetic stability of the BNYVV clone is not maintained when a high selection pressure is affecting the virus population in the resistant genotype. Although we confirmed the presence of each introduced mutation by conventional Sanger sequencing at the end of the experiments, it cannot be ruled out that spontaneous mutations outside the P25 ORF might manifested within the virus population. Nevertheless, the results presented herein underline that BNYVV still constitutes a severe threat to the sugar beet growers due to the rapid development of *Rz1* resistance-breaking strains. On the long term, the control of BNYVV will rely on the *Rz2* resistance gene that has been also introduced into sugar beet cultivars. The gene is located in close proximity to *Rz1* (Scholten et al., 1999), but appears to be based on a different mechanism (Scholten et al., 1994) that is more effective against BNYVV (Scholten et al., 1996). Although no resistance-breaking of *Rz2* has been reported yet, a continuous monitoring of the virus population is necessary. Our reverse genetic system constitutes a feasible tool to prove newly emerging mutations for their resistance-breaking ability.

## Data Availability Statement

The datasets generated for this study can be found in the EBI database PRJEB34221, NCBI MN148886, NCBI MN148887.

## Author Contributions

SL, EM, and MV planned and conducted the experiments. DW was responsible for NGS data analysis. SL wrote the manuscript with the collaboration of all authors.

## Funding

This research was funded by the Deutsche Forschungsgemeinschaft (project number 278522005).

## Conflict of Interest

The authors declare that the research was conducted in the absence of any commercial or financial relationships that could be construed as a potential conflict of interest.
