# Supplementary figures and images for: Application of a Reverse Genetic System for Beet Necrotic Yellow Vein Virus to Study Rz1 Resistance Response in Sugar Beet
Source: Front Plant Sci. 2020 Jan 17;10:1703. doi: 10.3389/fpls.2019.01703 (PMC6978805; doi:10.3389/fpls.2019.01703)

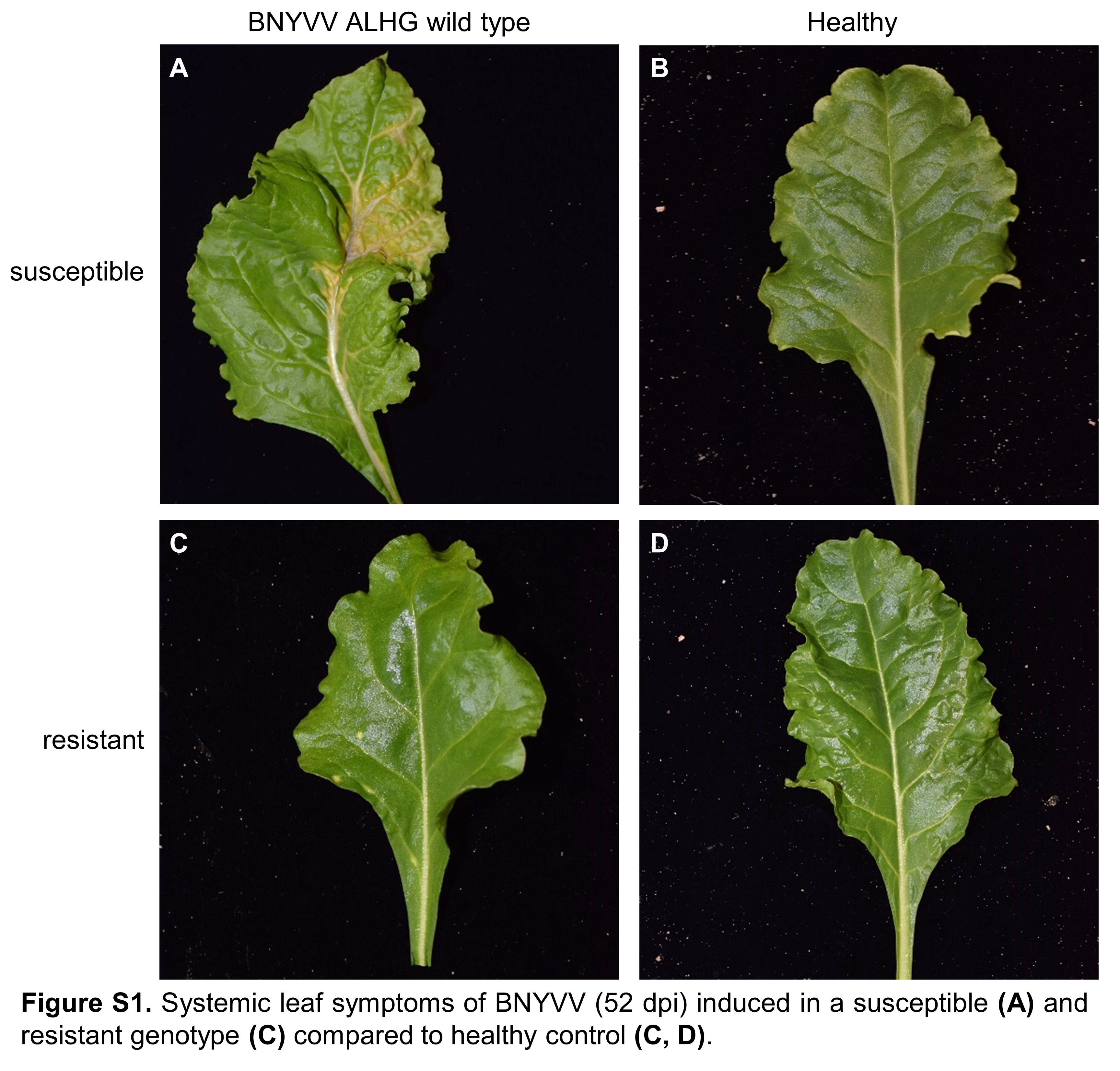

Supplement: Supplementary file 1 [file Image_1.jpeg]

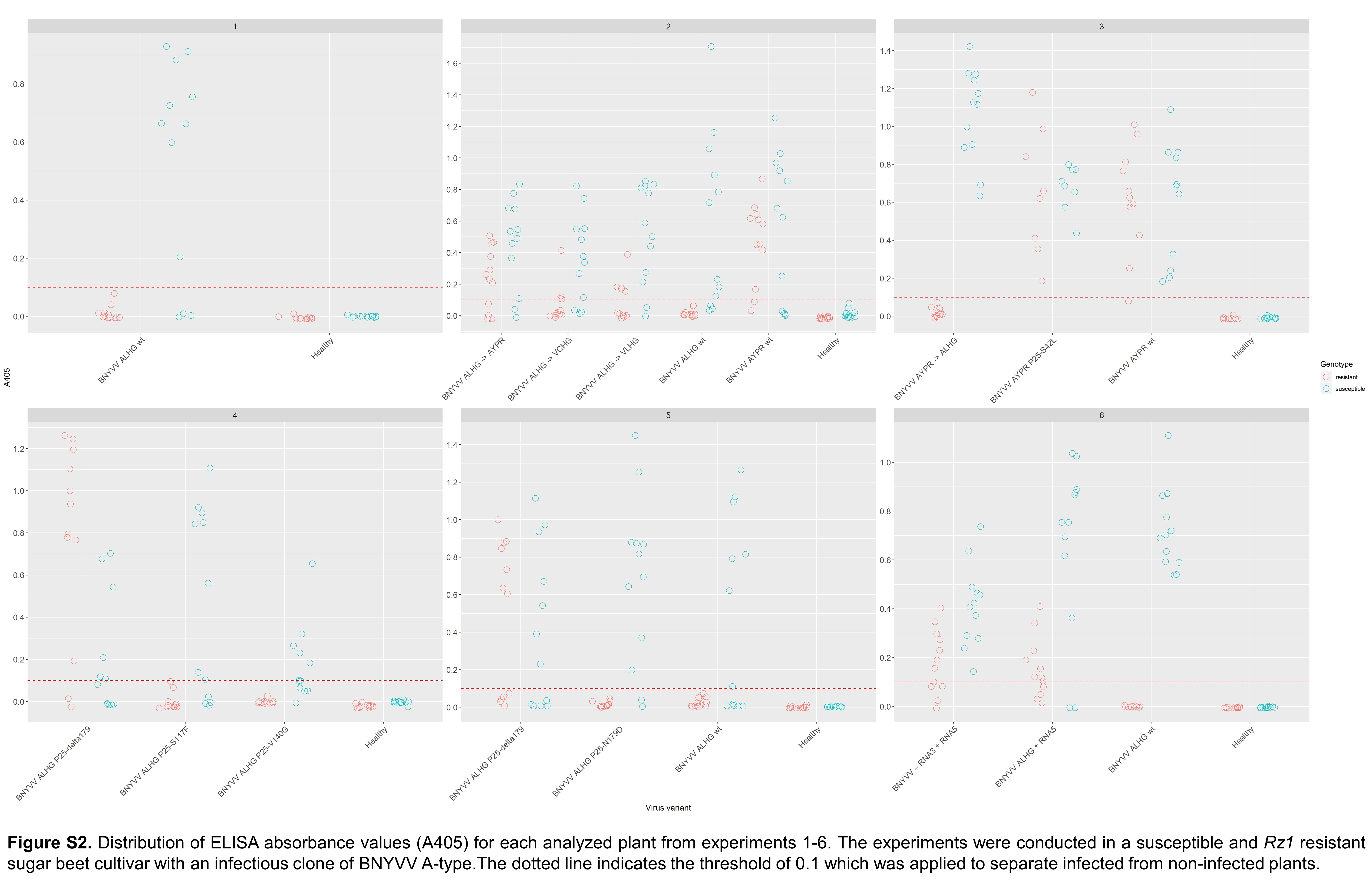

Supplement: Supplementary file 2 [file Image_2.jpeg]

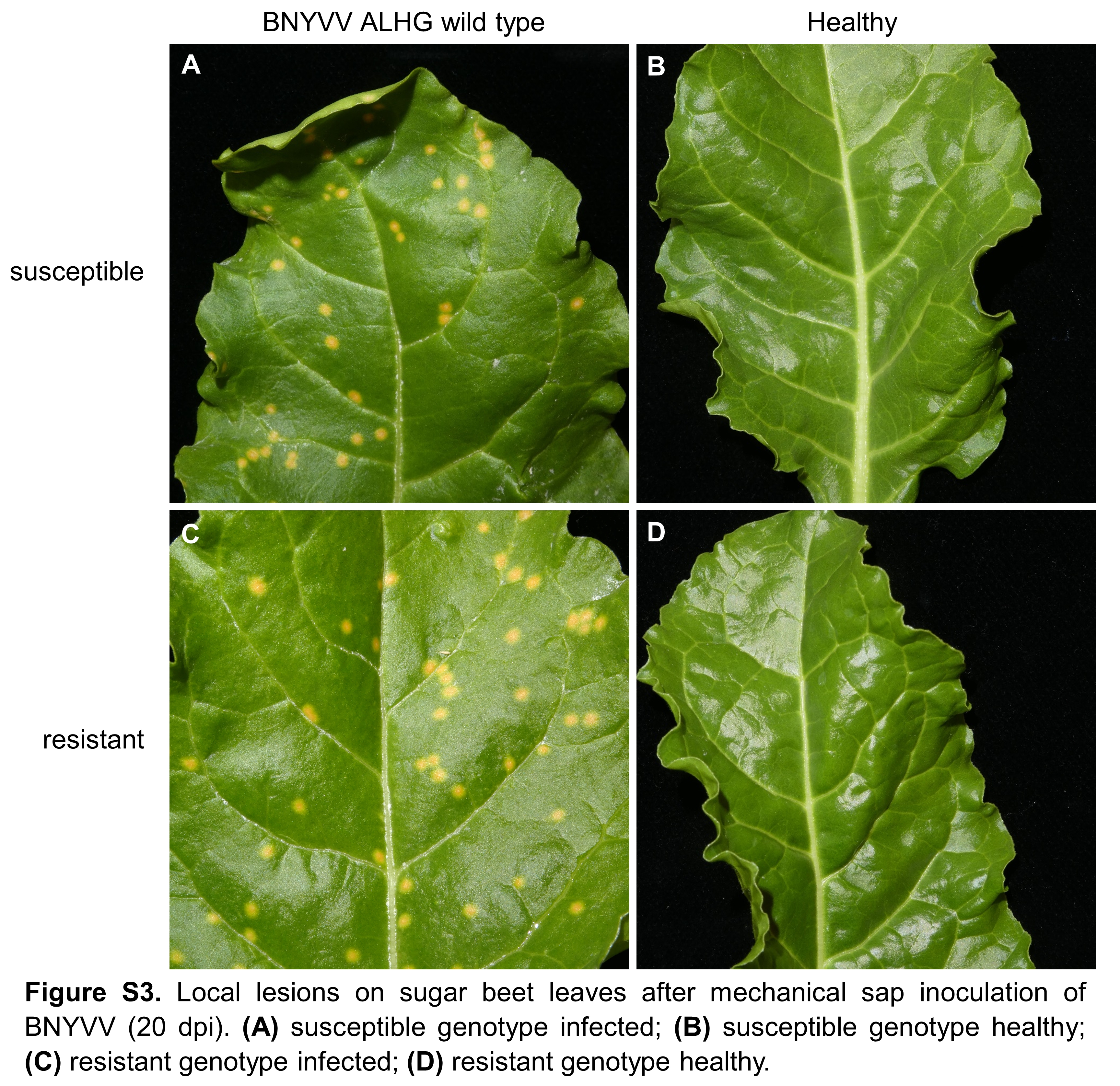

Supplement: Supplementary file 3 [file Image_3.jpeg]

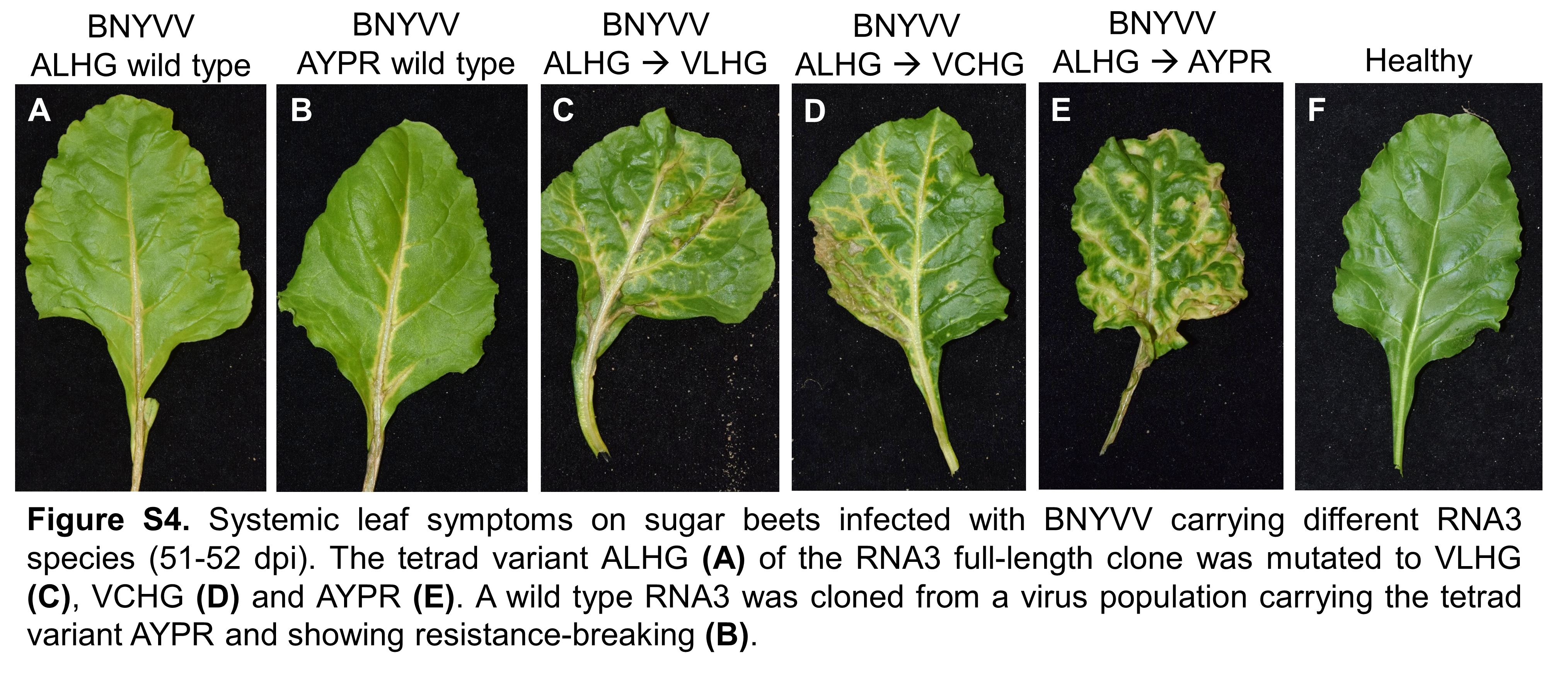

Supplement: Supplementary file 4 [file Image_4.jpeg]

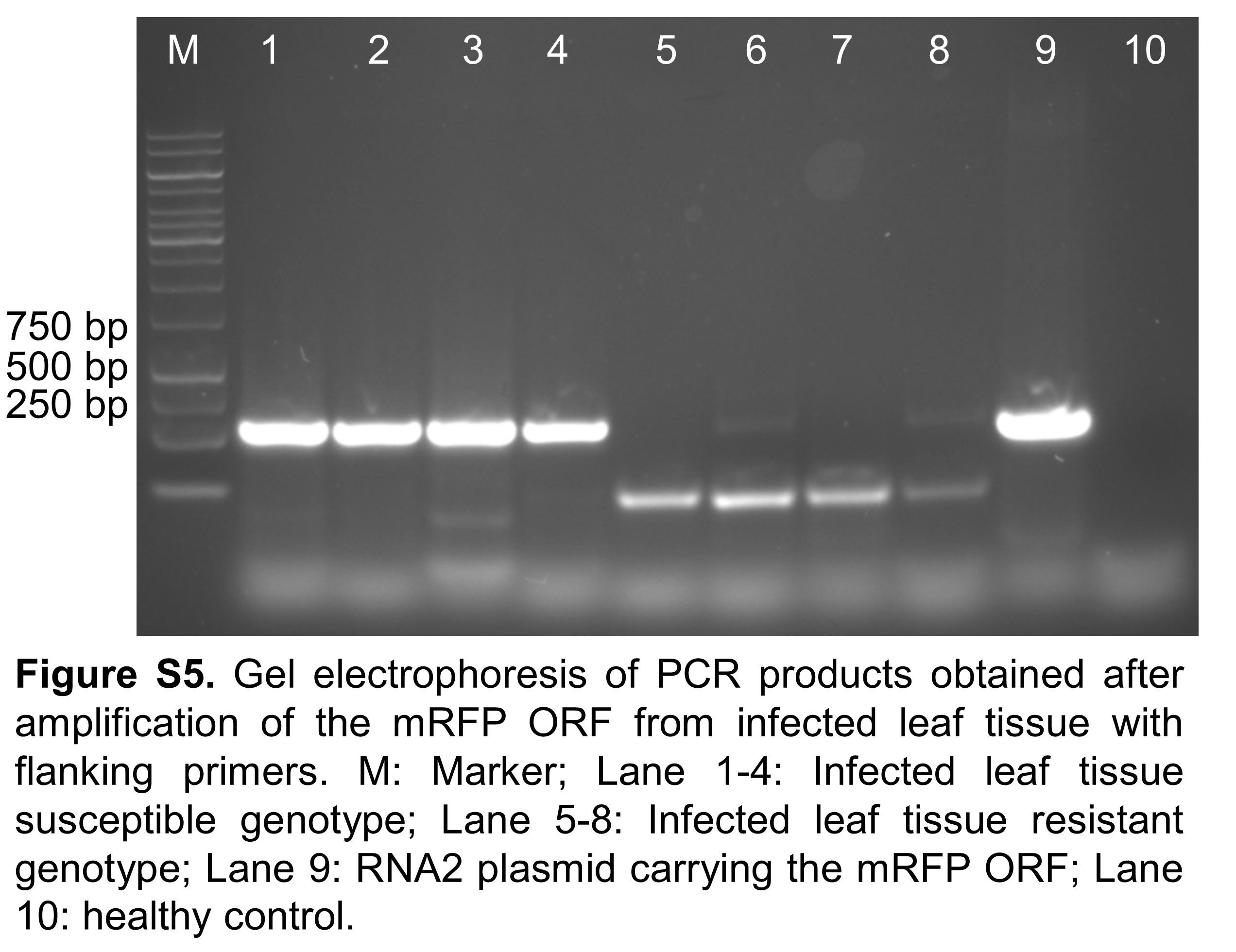

Supplement: Supplementary file 5 [file Image_5.jpeg]

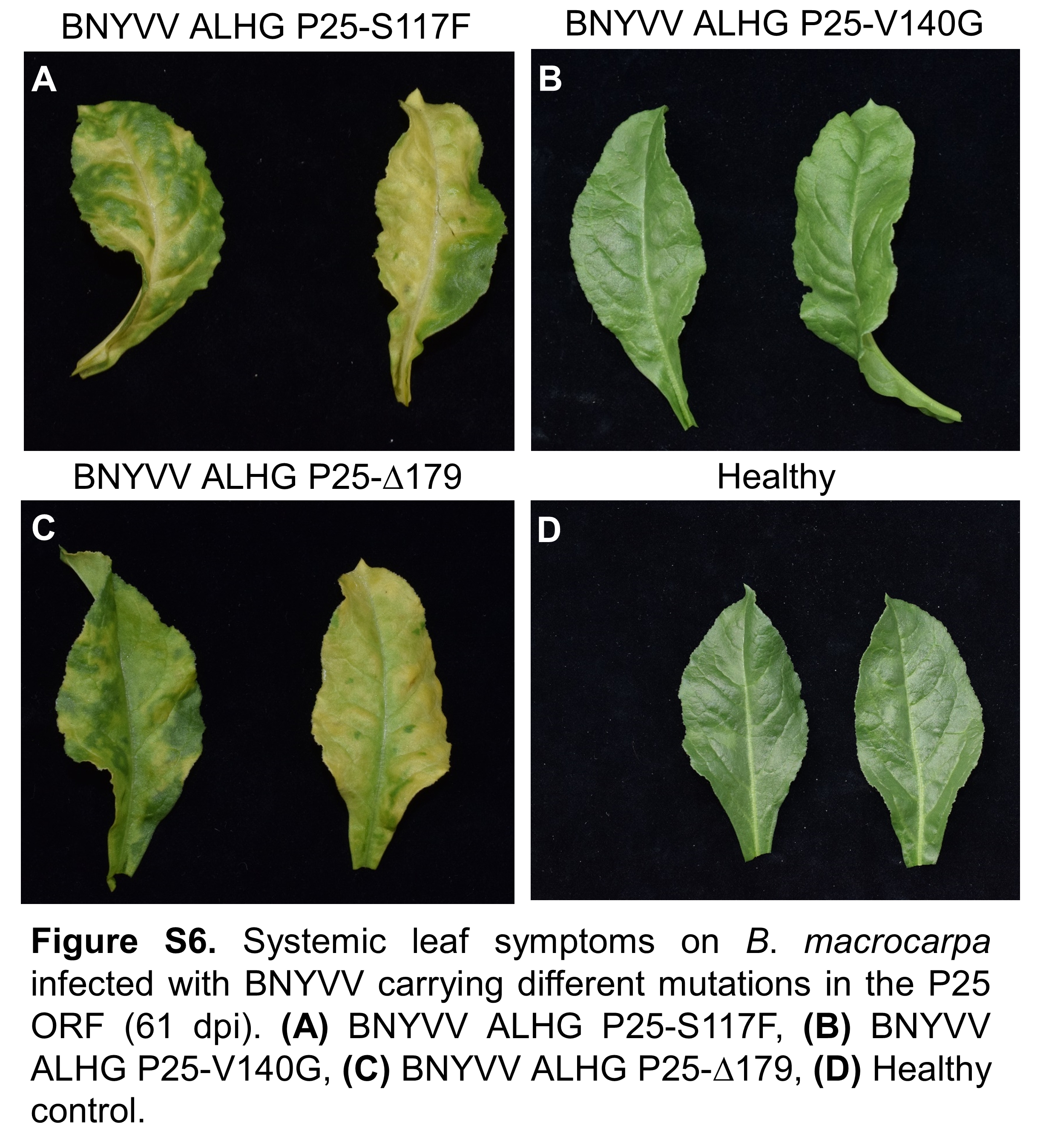

Supplement: Supplementary file 6 [file Image_6.jpeg]

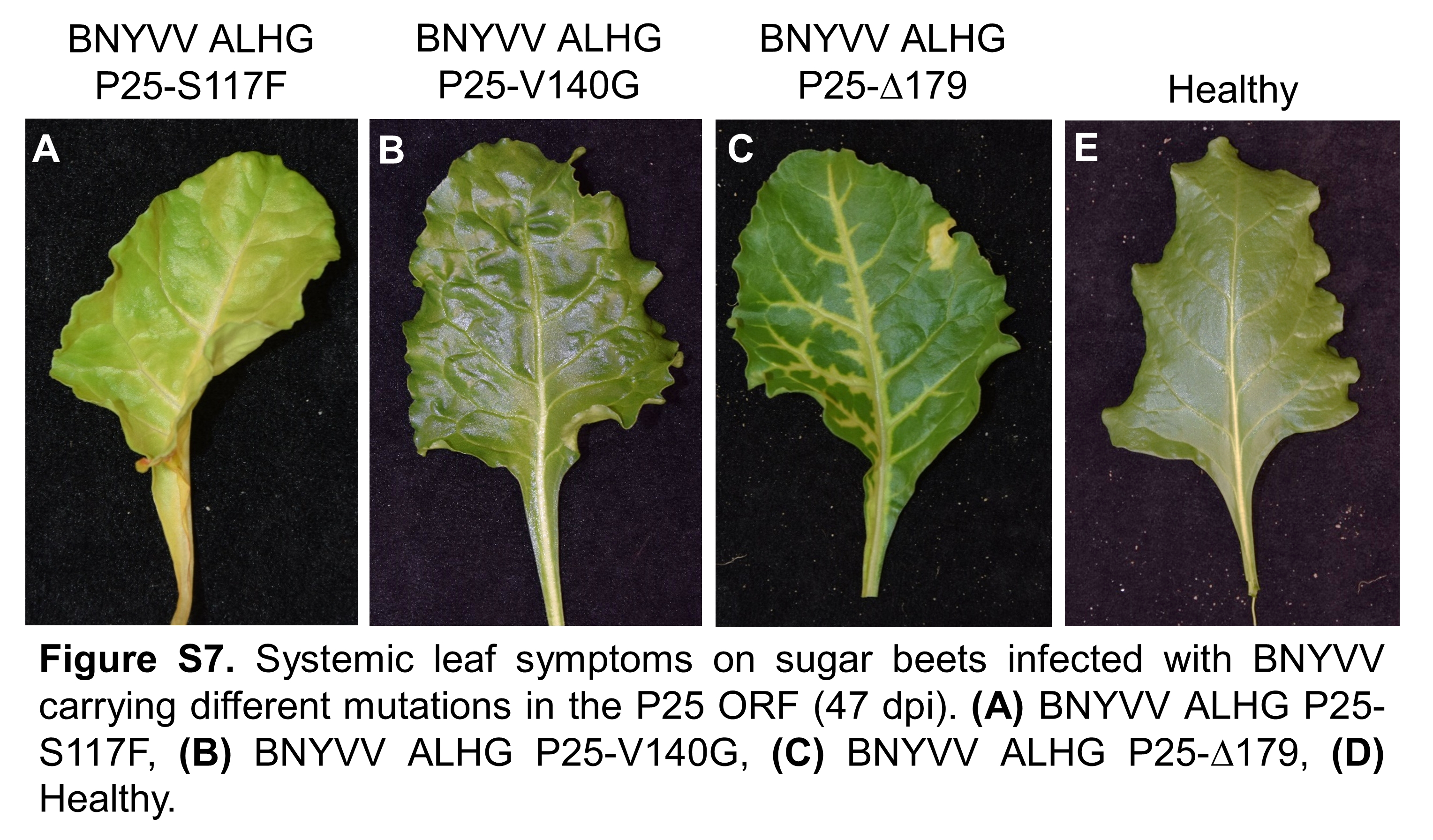

Supplement: Supplementary file 7 [file Image_7.jpeg]

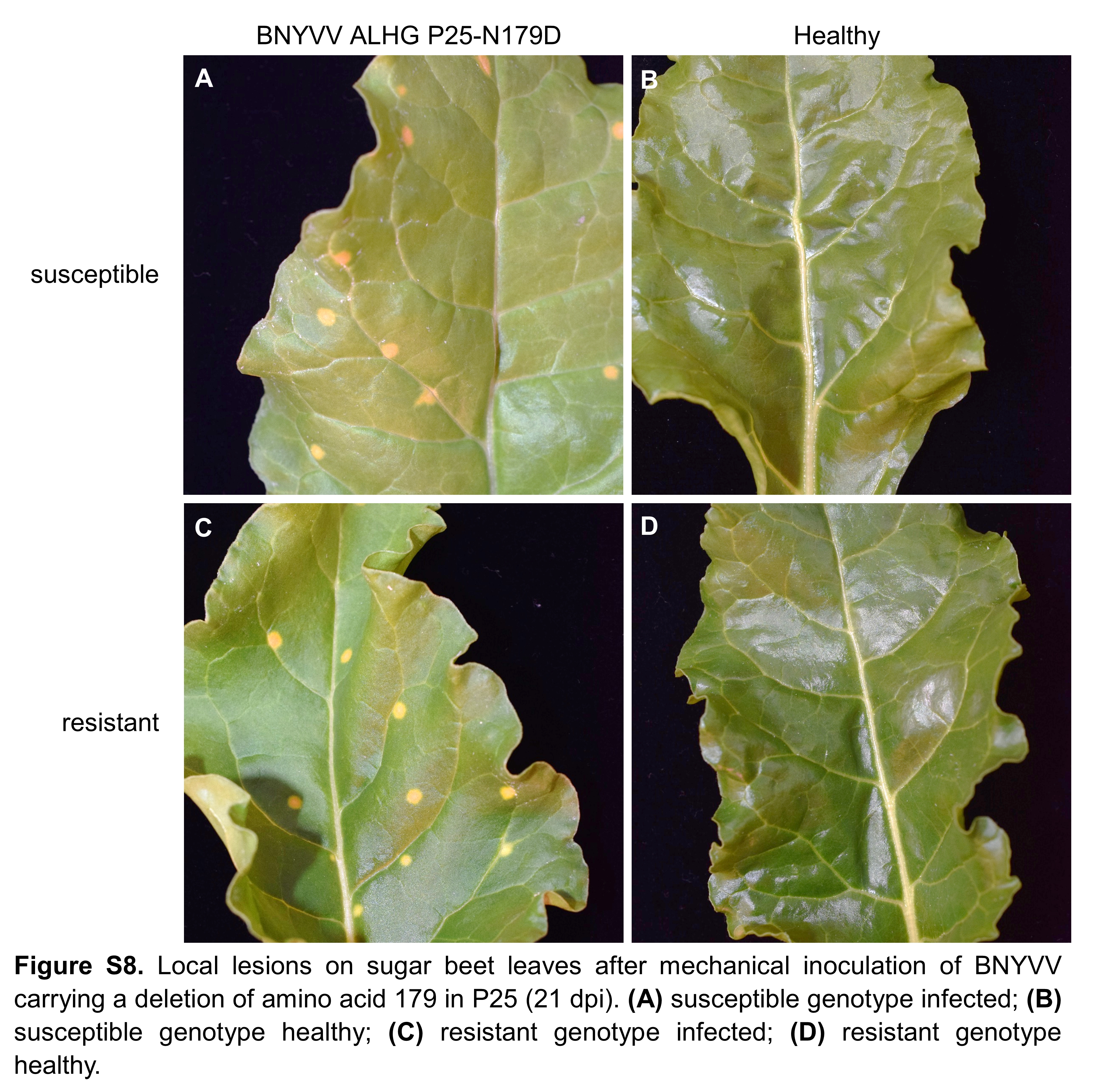

Supplement: Supplementary file 8 [file Image_8.jpeg]

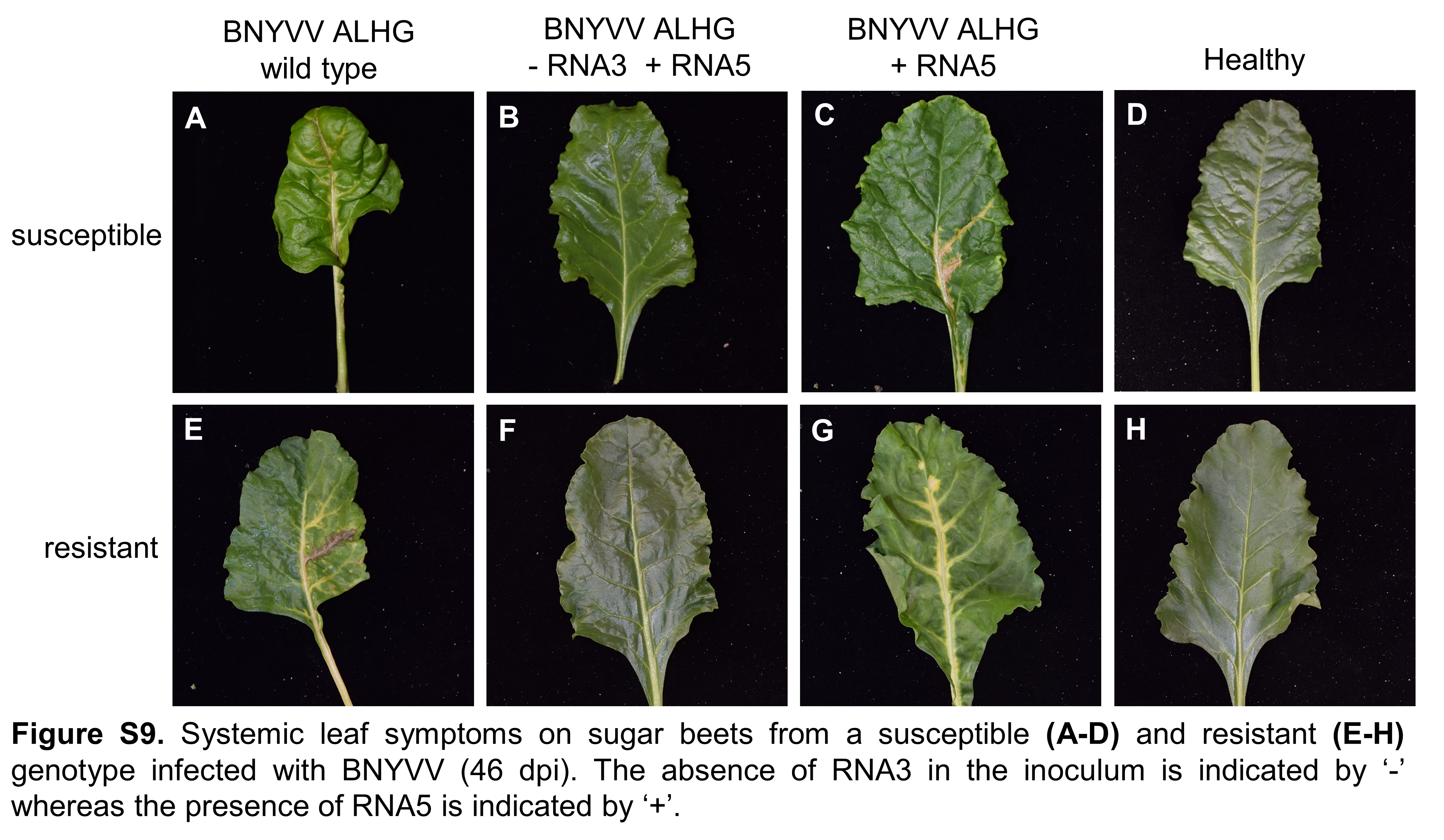

Supplement: Supplementary file 9 [file Image_9.jpeg]

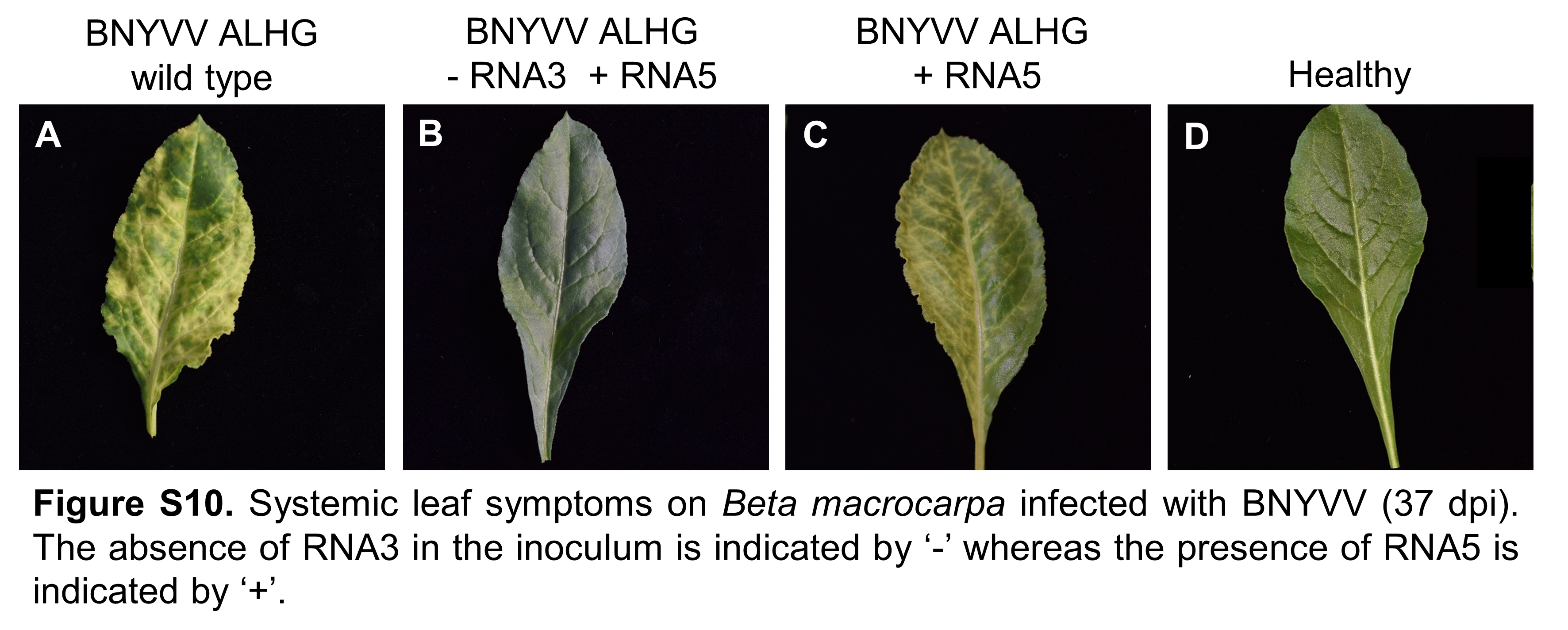

Supplement: Supplementary file 10 [file Image_10.jpeg]
