# Supplementary material for: Application of a Reverse Genetic System for Beet Necrotic Yellow Vein Virus to Study Rz1 Resistance Response in Sugar Beet
Source: Front Plant Sci. 2020 Jan 17;10:1703. doi: 10.3389/fpls.2019.01703 (PMC6978805; doi:10.3389/fpls.2019.01703)
Supplement: Supplementary file 11 [file Table_1.docx]

Supplementary Table S1. Overview of primers used in this study for PCR mutagenesis.

| **Primer name** | **Sequence (5’-3’)** | **Purpose** |
| --- | --- | --- |
| BN-P25-VLHG-fw | TCTTCATGGGCCTTATTGTG | Mutation of ALHG to VLHG  in the non-resistance-breaking BNYVV clone |
| BN-P25-VLHG-rv | ACACACAATAATCCACGAAACC |  |
| BN-P25- VCHG-fw | TCATGGGCCTTATTGTGGGTTTC | Mutation of ALHG to VCHG  in the non-resistance-breaking BNYVV clone |
| BN-P25- VCHG-rv | CAAACACACAATAATCCACGAAACCTT |  |
| BN-P25-AYPR-fw | GGCCTTATTGTGGGTTTC | Mutation of ALHG to AYPR  in the non-resistance-breaking BNYVV clone |
| BN-P25-AYPR-rv | TAGGATAAGCACACAATAATCCACGAAAC |  |
| BNYVV-RNA3up | AGGAAGTTCATTTCATTTGGAGAGGAAATTCAAAATTTACCATTACAT | Cloning of BNYVV RNA3 from a *Rz1* resistance-breaking strain into pDIVA. Overlapping vector sequence is underlined. |
| BNYVV-RNA3low | GAGATGCCATGCCGACCCTTTTTTTTTTTTTTTTTTTTTTGTCAATATAC |  |
| P25-AS42L/S-fw | TAA TCACGATAATCGTACTACACTG | Mutation of amino acid 42 from L to S in the wild type RNA3 derived from a resistance-breaking population. |
| P25-AS42L/S-rv | ACAAGTTAATAGCCCGACAGATAT |  |
| P25-AYPR/VLHG-fw | TTCTTCATGGGCCTTATTGTGGGTTTC | Mutation of AYPR to ALHG in the wild type RNA3 derived from a resistance-breaking population |
| P25-AYPR/ALHG-fw | CTCTTCATGGGCCTTATTGTGGGTTTC |  |
| Seq-p25-Tetrade | ATTCTTAGCACACATGGTAA | Sequencing primer for P25 |
| P25-seq-fw | ACACTCTTTCCCTACACGACGCTCTTCCGATCTTATCTGTCGGGCTATTAACT | Primers for the first PCR to prepare amplicons for deep sequencing. Adaptor sequence is underlined |
| P25-seq-rv | GTGACTGGAGTTCAGACGTGTGCTCTTCCGATCCATCACCTGTATACAAACCA |  |
| D501fw | AATGATACGGCGACCACCGAGATCTACACTATAGCCTACACTCTTTCCCTAC | Primers for the second PCR to prepare amplicons for deep sequencing. The index for barcoding is underlined |
| D701rv | CAAGCAGAAGACGGCATACGAGATATTACTCGGTGACTGGAGTTCAGa |  |
| D702rv | CAAGCAGAAGACGGCATACGAGATTCCGGAGAGTGACTGGAGTTCAG |  |
| D703rv | CAAGCAGAAGACGGCATACGAGATCGCTCATTGTGACTGGAGTTCAG |  |
| D704rv | CAAGCAGAAGACGGCATACGAGATGAGATTCCGTGACTGGAGTTCAG |  |
| D705rv | CAAGCAGAAGACGGCATACGAGATATTCAGAAGTGACTGGAGTTCAG |  |
| D706rv | CAAGCAGAAGACGGCATACGAGATGAATTCGTGTGACTGGAGTTCAG |  |
| D707rv | CAAGCAGAAGACGGCATACGAGATCTGAAGCTGTGACTGGAGTTCAG |  |
| D708rv | CAAGCAGAAGACGGCATACGAGATTAATGCGCGTGACTGGAGTTCAG |  |
| D709rv | CAAGCAGAAGACGGCATACGAGATCGGCTATGGTGACTGGAGTTCAG |  |
| P25-AS179-N/D-fw | GATTGTGTGTTACGTTTTGAGTCC | Mutation of amino acid N179D |
| P25-AS179-N/D-rv | ACCGTGTTTACCAAACACACC |  |
|  |  |  |
| p25CtoTs | GAGTTGCTGGTTTTTAATGGTCTCGTCATC | Mutation amino acid S117F |
| p25CtoTas | GATGACGAGACCATTAAAAACCAGCAACTC |  |
| p25TtoGs | GATGGTTTTGAAGTTGGAGATTTCACGACTGTC | Mutation amino acid V140G |
| p25TtoGas | GACAGTCGTGAAATCTCCAACTTCAAAACCATC |  |
| p25DAsps | GTTTGGTAAACACGGTTTGTGTGTTACGTTTTGAG | Deletion of amino acid 179 (Δ179) |
| p25DAspas | CTCAAAACGTAACACACAAACCGTGTTTACCAAAC |  |
| BNYVV-RNA5-up | AGGAAGTTCATTTCATTTGGAGAGGAAATTCAAAGTACTTTCATATTG | Cloning of BNYVV RNA5 from P-type into pDIVA. Overlapping vector sequence is underlined |
| BNYVV-RNA5-low | GAGATGCCATGCCGACCCTTTTTTTTTTTTTTTTTTTTTTTGTCAATACA |  |
